# Death in Advance? A critique of the “Zombification” of people with dementia

**DOI:** 10.1007/s40656-022-00512-z

**Published:** 2022-08-16

**Authors:** Mark Schweda, Karin Jongsma

**Affiliations:** 1 Division of Medical Ethics, Department of Health Services Research, School for Medicine and Health Sciences, University of Oldenburg, Oldenburg, Germany; 2 Department of Medical Humanities , UMC Utrecht, Utrecht, The Netherlands

**Keywords:** Dementia, Life course, Illness metaphors, Moral reasoning, Regime of living

## Abstract

This contribution sets out to criticize the prominent metaphor of “death while alive” in the context of dementia. We first explain the historical origin and development as well as the philosophical premises of the image. We then take a closer look at its implications for understanding dementia and societal attitudes and behaviours towards those affected. In doing so, we adopt a life course perspective that seeks to account for the ethical significance of the temporal extension and structure of human life. According to this perspective, individual existence in time is characterized by normative standards of age-appropriate behavior, evaluative standards of a good life, and teleological notions of successful development which require theoretical analysis and ethical discussion. Such a perspective can contribute significantly to spelling out the implications of the metaphor of death while alive and to criticizing their problematic aspects. Indeed, it makes clear that this metaphor aligns dementia with a different point in the human life course, thus ultimately framing it as a kind of deviation from the biographical norm, a disruption in an assumed temporal order of existence. At the same time, the life course perspective can help to understand why this conception involves ethically problematic distortions and blind spots. The resulting considerations allow conclusions with regard to medical and care ethical debates about self-determination, surrogate decision making, and advance directives in the context of dementia. Furthermore, on a theoretical-conceptual level, they also illustrate the importance of a biography- and culture-sensitive approach to philosophical and ethical reasoning in biomedicine and the life sciences.

**Introduction**.

“Dementia is a living death for 700,000 Britons” (Hill, 2008), read a headline of an article in the British newspaper *The Guardian*. Starting from an emotional description of the fate of a middle-aged husband and father with early-onset Alzheimer’s disease, the text goes on to describe the severe individual and societal consequences of dementia: “To be trapped in the ‘living death’ of dementia is, for many, the most fearful of all endings. Relatives of sufferers often describe it as the illness that slowly switches off the lights in the brain. Savagely and pitilessly, it strips away memory, language and personality, leaving only the shell of its victims behind. Finally, it robs them of their lives” (ibid.).

As this example illustrates, the idea of being dead while still alive plays a significant role in contemporary discourses on dementia, just like its prominent counterpart, the notion of dementia as a “return to childhood” (Jongsma & Schweda, 2018). In general, our view of dementia seems almost inescapably shaped by such time-honoured and powerful cultural tropes, that is, recurring forms of figurative language. Especially metaphors play an important role in this context (Zeilig, 2013). By drawing analogies to a familiar realm of experience, they enable us to develop an understanding of an otherwise elusive and ultimately unfathomable process. After all, those affected by dementia gradually lose the ability to express their own subjective perspective and experience. This makes their inner world increasingly inaccessible and enigmatic from an external point of view.

At the same time, however, it is important to realize that this metaphorical framing of dementia is anything but innocent (Van Gorp & Vercruysse, 2012). The figurative sphere has its own internal structure and logic beyond the intended analogy and point of comparison. This metaphorical “excess of meaning” (Blumenberg, 2010) can effectively override the literal, factual domain and lead to inappropriate conceptions of the issue at hand. Accordingly, dementia metaphors can have far-reaching morally as well as politically problematic consequences for our view of the phenomenon itself and the way we treat those affected. Thus, they can compromise their self-understanding and undermine our respect for their personhood, self-determination, and even fundamental human rights (Jongsma & Schweda, 2018; Zimmermann, 2017).

This contribution aims to criticise the powerful metaphor of “death while alive” in the context of dementia.^1^ We first explain the historical origin and development as well as the philosophical premises of the analogy. We then take a closer look at its implications for common understandings of dementia and societal attitudes and behaviours towards those affected. In doing so, we adopt a life course perspective that seeks to account for the ethical significance of the temporal extension and structure of human life. According to this perspective, individual existence in time is characterized by normative standards of age-appropriate behaviour, evaluative measures of ageing well, and teleological notions of successful development whose implications must be analysed and discussed (Schweda, 2017). Such a perspective can contribute significantly to spelling out the moral implications of the metaphor of death while alive and to highlighting its shortcomings. As we will argue, this metaphor assimilates dementia to a different point in the human life course, thus ultimately framing it as a “death in advance”, that is, a kind of deviation from the biographical norm, a disruption in an assumed temporal order and sequence of existence. At the same time, the life course perspective can help to understand why this viewpoint involves problematic distortions and blind spots. The outlined considerations allow conclusions with regard to medical and care ethical debates about self-determination, surrogate decision making, and advance directives in the context of dementia. Furthermore, on a theoretical-conceptual level, they also illustrate the importance of a biography- and culture-sensitive approach to philosophical and ethical reasoning in the context of the life sciences.

## Background: the “zombification” of people with dementia

In contrast to the image of a “second childhood”, the metaphor of “death while alive” usually refers to the advanced stages of senile dementia. In ancient mythology, philosophy, and medicine, individuals with reduced or extinguished consciousness were sometimes associated with ideas of an intermediate somnambulistic state, a kind of shadowy realm between animation and inanimate matter, life and death (Gundert, 2000). To this day, popular culture is familiar with legends of the undead and revenants. In contrast to ghosts, these are attributed a purely physical, corporeal mode of being, devoid of anything spiritual, psychological, or mental (Keyworth, 2020).

On a larger scale, however, the death metaphor for senile dementia only became relevant in the second half of the 20th century. The establishment of modern intensive care units in the 1970s opened novel technical possibilities for maintaining vital body functions in the event of complete and permanent loss of consciousness, thus increasingly blurring the clear demarcation between life and death. This gave rise to discussions about the ontological, biological, and moral status of comatose and brain-dead people. Categories of gradual and diminished vitality were formulated, for example, in analogy to the plant world in the sense of a merely vegetative existence (Wijdicks, 2021; Brukamp, 2012).

Another prominent cultural point of reference was the notion of the “zombie”. As a modern version of the living dead, the zombie has become an iconic figure in contemporary popular culture in horror films like George A. Romero’s genre-defining *Night of the Living Dead* or successful television series, such as *The Walking Dead* (Travis, 2015). In the wake of the popularization of the dementia discourse, titles referring to corresponding ideas of liminal existence and death while alive also began to appear in the pertinent popular advice and self-help literature of the 1980s, such as *Alzheimer’s Disease: Coping with a Living Death* (Woods, 1989) or *The Living Dead: Alzheimer’s in America* (Lushin, 1990).

Ultimately, the basis of the living death-metaphor is a particular view of human nature, a dualist understanding rooted in the Platonic tradition and also dominating Cartesian thought, which takes the strict distinction between rational consciousness and a transient and problematic physical-material body as the constitutive trait of human existence. According to this view, the characteristic that defines the human being as such and distinguishes it from other living beings is its capacity for reasoning and rational self-knowledge, self-determination, and life planning. Along these lines, the mentalist paradigm and epistemological perspective of the philosophy of Enlightenment still defined the core of personhood in terms of self-consciousness and tied personal identity to the ability to remember and to establish inner continuity by linking mental states (Locke, 1964 [1690], p.211). In this mentalist and rationalist frame of reference, a life that does not (or no longer) possess these capacities cannot be regarded as a human life in the full sense of the word (for a critique, see Hughes, 2001). Accordingly, the loss of cognitive capacities, especially self-consciousness and memory, appears as tantamount to the loss or demise of the self as such, and thus as the end of personhood, of truly human existence (for a critique, see Post 2000).

Understood this way, the notion of a death while alive was rather common in discourses on dementia until the 1980 and 1990 s. In book titles such as *Death in Slow Motion* (Cooney, 2004), or *A Curious Kind of Widow: Loving a Man with Advanced Alzheimer’s* (Davidson, 2006), the pertinent background imagery clearly shines through. The death metaphor has also found widespread dissemination in newspaper articles and print media reports on dementia (e.g., Clarke 2006; Kirkman, 2006). In fact, it still pervades many contemporary media representations and public discussions Peel, 2014; Johnstone, 2013; van Gorp & Vercruysse 2012). In 2011, for example, U.S. preacher and former contender for the Republican presidential nomination Pat Robertson declared on his television program that it was morally acceptable to divorce a spouse suffering from Alzheimer’s disease because the disease was akin to a kind of death (Eckholm, 2011). Up until today, traces of the pertinent notions, comparisons, and assimilations of dementia, dying, and death can be found frequently in print and online media discourses as well as in movies and literary fiction (Kleinke, 2022; Sm-Rahman et al., 2021; Low & Purwaningrum, 2020).

However, the metaphor of death while alive is by no means limited to the domain of popular culture. It has also permeated and shaped scientific expert discourse as well as professional medical and nursing practice in the context of dementia for decades. Especially in the earlier phases of the academic discussion, Alzheimer’s dementia was often described as a “death in life” (Kastenbaum, 1988) or “death before death” (Cohen & Eisdorfer, 1986). In fact, for a long time, it was not uncommon in medicine, nursing research, and the health sciences to deny people with dementia any subjective inner life. It was assumed that all the disease left was an inner “void” (Phinney & Chesla, 2003, p.292), an “empty shell” that was “without content” (Fontana & Smith, 1989, p.36). Accordingly, dementia care has repeatedly been described as care for the “living dead” (Baher, 2004; Dunkle, 1995). Affected patients were frequently not informed about their diagnosis and usually not involved in decision making processes regarding their own medical treatment (Pinner & Bouman, 2002). In medical ethical literature, they were occasionally even classified as “non-persons” or “post-persons” who might no longer be accorded the same moral status and the same rights as all other people (Brock & Buchanan, 1990, pp.152–189).

Against this backdrop, a cultural “zombification” of people with dementia has been observed, encompassing manifold associations between the symptoms and the societal consequences of neurodegenerative diseases and the uncanny notion of an army of mindless, insentient, and menacing living corpses consuming our livelihood (Behuniak, 2011). For example, the pertinent metaphorical framing projects images of a loss of identity and humanity, a failure to recognize others, to function as a familiar social counterpart, and to conform with acknowledged societal expectations, as well as apocalyptic demographic scenarios of a population-wide plague of epidemic proportions and devastating cultural as well as socio-economic consequences (ibid., pp.77–85). Of course, the metaphor of the living death may also reflect a coping strategy in the context of anticipatory loss and grief of caring relatives and thus does not automatically imply a “zombification” of people with dementia (Sweeting, 1991). Nevertheless, the use of the imagery runs the risk of actualizing and reinforcing a widespread cultural interpretation of the syndrome that makes those affected appear as insentient creatures that represent a threat to their immediate environment and a burden to society as a whole (Behuniak, 2011).

## Anthropological inadequacy

At closer inspection, it becomes clear that the metaphor of “death while still alive” is based on a series of problematic assumptions. The equation of dementia and death presupposes a reductionist, one-sided mentalist and intellectualist conception of being human which links truly human existence as personhood to higher cognitive abilities and thus degrades or excludes individuals who do not (or no longer) possess these abilities (for a critique, see Spaemann, 2006). To the extent that the concept of personhood also denotes a moral or legal status, such an exclusive conception can have fundamental and far-reaching ethical consequences. This becomes particularly evident in the aforementioned positions characterizing people with dementia as ‘non-persons’ or ‘post-persons’ who no longer deserve the same level of respect or are denied equal rights (for a fundamental critique, see Higgs & Gilleard, 2016).

In fact, the critique of this reductionist anthropological view emerged not least from the so-called personhood movement in dementia studies. The respective scholars emphasized that personhood and personal identity are not just a matter of inner mental states and their continuity in the sense that they simply “evaporate” as soon as memory declines. According to them, personhood and personal identity are instead always embodied and socially situated (Hutmacher, 2021; Hughes, 2001). Embodiment means the “sedimentation” of an individual’s lived life and personality, with its characteristic temperament, preferences and behaviours, in a set of distinctive facial expressions, inflections, gestures, physical postures, habits, preferences and aversions, and so on. These can outlast the loss of memory and higher cognitive capacities and thus keep the person present (Fuchs, 2020). Social situatedness, in turn, refers to the relational dimension of personal existence, the fact that personal identity and biographical continuity are not only based on inner mental functions, but also depend on the recognition, support and participation of others. We cannot simply define who we once were and now are in a solitary inner act of consciousness. Our identity is also negotiated - and occasionally challenged - in close social relationships and acts of (mutual) recognition. These are factors that can also keep an individual’s personal identity present long after his or her conscious self-reflection and memory have expired (Tolhurst et al., 2017).

## (Bio-)medical inadequacy

Apart from the reductionist view of human existence, the metaphor of death while alive is also based on a narrow neurocognitivist understanding of dementia itself. The disease process is only considered to the extent that it affects the level of neurophysiological functions and cognitive performance. Physical, psychological, and social aspects of dementia tend to be ignored or at least strongly neglected, which impedes an appropriate understanding of the syndrome as such and makes it difficult to deal with those affected in an adequate way (Lyman, 1989).

In addition, the narrow neurocognitivist perspective reinforces an exceptionally negative image of dementia. By focusing on a domain in which the disease process can hardly be described other than as a progressive loss and deterioration of abilities, it supports a one-sided deficit-oriented view of dementia which systematically excludes possible positive experiences such as increased emotional sensitivity or a rapprochement in the relationship. This ultimately manifests itself in the equation of the disease with dying and death (Aquilina & Hughes, 2006).

However, even if one were to adopt a neurocognitivist approach to dementia, the analogy with dying and death would be an inadequate description of the disease process. In most cases, dementia does not lead to a complete loss of consciousness, as it is assumed for some comatose and all brain-dead individuals. People with dementia are normally still somewhat aware of their surroundings and generally also react to external stimuli. The range of possible interactions seems to depend to a considerable extent on the attention, empathy, and approach of the social environment. For example, there are quite a number of impressive testimonies for the potential of music and theatre therapy approaches to elicit vivid expressions of inner life even from people with advanced dementia and to open up possibilities for extremely lively interaction and communication (Kontos, 2014; Kontos et al., 2017).

Moreover, in cases of Alzheimer’s dementia in particular, there are numerous reports of remarkably lucid episodes up to the very last stages of the disease, episodes in which those affected are suddenly much more mentally acute and present and can sometimes even articulate their own experience of the disease and of their precarious situation (Normann et al., 2006). At least with regard to such reports of limited expressive possibilities, the image of an individual being trapped in the disintegrating neuronal structures of his or her brain may actually appear more appropriate – and also suggests a different, more compassionate attitude and behaviour vis-a-vis those affected – than the metaphor of death in the sense of a complete and irreversible disappearance of the person as such (Aquilina & Hughes, 2006, p.145).

## Biographical inadequacy

The inappropriateness of the comparison between dementia and death becomes particularly clear from a life course-perspective. According to this approach originally formulated in developmental psychology and the sociology of ageing, individual existence in time is not just a continuous biological process. Instead, it is also shaped by a variety of normative standards of age-appropriate behaviour, evaluative measures of ageing well, and teleological conceptions of human development which ethics has to make explicit and discuss (Schweda, 2017).

From a life course perspective, we can see that the metaphor of the living death provides an interpretation of dementia by analogizing it to another point in the temporal course of human life. As it starts from a dualist conception of personhood based on higher cognitive capacities, the image suggests that the decline of these capacities is tantamount to the end of human life in the full sense, i.e. the death of the person. All that remains is a body that only bears an external resemblance to the former person, an “empty shell” or a “living corpse” (Behuniak, 2011). In this sense, dementia is framed as a “death in advance”, a deviation from the biographical norm and a disruption in the assumed order and sequence of human existence in time.

While the counterpart metaphor of the “second childhood” tends to obliterate the moral significance of the past and especially of the past life history of those affected, the death metaphor thus annihilates their present and future. Ultimately, it ignores the entire present condition and future fate of the affected individual as morally irrelevant. In doing so, it fails to recognise that the lives of those affected are by no means over. In fact, the invalidation of the present and future seems particularly perfidious and alarming, since this present and future life phase of dementia is characterised by an increased vulnerability and thus precisely by the need for special moral sensitivity, consideration, and protection (Petherbridge, 2019). Instead of taking this vulnerability into account, the death metaphor presents dementia as a morally indifferent phenomenon, indeed, ultimately as the biographical irregularity of a “living death” that can only be rectified by the complete demise of the body as well.

## Detrimental practical consequences

Whereas the image of the “return to childhood” may in many cases reflect rather well-meaning intentions towards people with dementia, the metaphor of “death while alive” primarily expresses deeply negative attitudes essentially marked by fear, loathing and even contempt. In fact, it promotes a view of dementia that facilitates and promotes the degradation of those affected and undermines their legal, moral and political status. In this regard, the theoretical stigmatisation through the death metaphor corresponds to long-standing practical experiences of disregard, neglect, and discrimination of people with dementia in everyday life, in the healthcare context, as well as on a more general sociocultural level.

In everyday life, the overall view of dementia that the imagery of death suggests seems to have many parallels to a “social death” (Sweeting & Gilhooly, 1997) that those affected frequently experience before their actual physical end. They are no longer perceived and treated as a valuable social counterpart and interaction partner but are instead ignored and rejected (Brannelly, 2011). Their perspectives and interests as well as their verbal or non-verbal expressions are disqualified as valid contributions to rational discourse and excluded from public and political representation (Jongsma et al., 2017). Ultimately, these tendencies amount to a general cultural inclination to “other” very old age and especially dementia as its pivotal symbol as a form of the “abject” and ban those affected from public view and social participation (Gilleard & Higgs, 2015). This stigma is known to reinforce self-stigmatization and adds to the psychological burden of caring relatives of people with dementia (Price & Hill, 2021).

With regard to medicine and healthcare, empirical studies have shown that professional caregivers show less empathy with their patients with dementia, communicate and interact less with them, and provide them with overall poorer care when they do not ascribe these patients an inner life of their own (Digby et al., 2016; Hunter et al., 2013; Ekman et al., 1991). Accordingly, people with dementia have been and still are particularly vulnerable to neglect, disregard, and abuse in hospitals and institutional care settings (Kelly & Innes, 2013). For example, they frequently experience restrictions of fundamental dimensions of their freedom through coercive measures such as physical restraints or antipsychotic drugs (Pu & Moyle, 2020). As a consequence, they often have an increased risk of morbidity and mortality than older patients without cognitive impairments (Ralph & Espinet, 2018). Recent commentaries have even drawn connections between the metaphorical imagery of living death and the high mortality rates of people with dementia in long-term care facilities during the COVID-19-pandemic (Kontos et al., 2021).

On a sociocultural level, the horrifying idea of being dead while still alive can also fuel and reinforce exaggerated fears of dementia which may sometimes even motivate rash decisions, e.g., in favour of pre-emptive suicide, when a diagnosis is made (Benbow & Jolley, 2012). Media studies have analysed how framing Alzheimer’s disease through the death metaphor has been instrumentalised in public bioethical debates as a rhetorical device for promoting a liberalisation of active euthanasia without further arguments (Johnstone, 2013). In fact, in the style of self-help guides, recent publications fuel abhorrence of dementia while at the same time providing hands-on recommendations on how to obtain “active deliverance” (Brewer, 2019). Furthermore, in the debate on how to cope with rising healthcare costs in ageing societies and the accompanying proposals for age-based rationing of healthcare resources, it has been suggested that older people with cognitive impairments are regularly no longer regarded as persons but only as a socio-economic burden. This dehumanizing perspective excludes them from the moral and legal community and degrades them to “bare life” stripped of any normative significance, which puts the protection of their dignity and right to life at risk (Burke, 2019; Kaufman, 2006; Leibing, 2006).

## Conclusions: against “zombification”

Metaphors play an important role in the cultural interpretation of reality. By reducing complexity and drawing analogies to familiar domains, they facilitate the reflection and comprehension of abstract, complex phenomena that are difficult to grasp otherwise. This also applies to the metaphors used in the cultural interpretation of dementia. It is remarkable that the metaphor of the “living death” – just like the other prominent image in this context, the “return to childhood” – opens a temporal perspective and frame of reference. It ultimately implies that dementia is somehow a temporal aberration, a “death in advance”, and that those affected deviate from the presumed biographical order of human life. While the childhood metaphor suggests a reversal of the normal life course, the death metaphor rather alludes to notions of fast-forward, acceleration, and prematureness.

Although the trope may have different functions in different practical contexts, for example a consolidating function in cases of anticipatory loss and grief of family carers, the underlying notion of dementia in terms of death in advance appears highly problematic on several levels. It is based on a narrow perspective that neglects the important role of embodiment, personal development, and social connectedness in the context of dementia. As a result, it also overlooks the ethical significance of the life course and the fact that personal identity and autonomy emerge and change over time, with individuals developing and continuing a unified life story. The resulting reductionist understanding of dementia promotes a “zombification” of those affected that can lead to disregard and cause harm. Just as their past history cannot be ignored, neither can their present demeanour and future destiny. The journey of people with dementia cannot be adequately understood in terms of a return to the beginning or a premature end. It inevitably continues into the future.

Our analysis also allows a number of conclusions with regard to the management of dementia in everyday life as well as in medical research and care. A first point concerns communication, interaction, and decision-making processes. The metaphor of “living death” tends to frame people with dementia as passive, unresponsive agents without subjectivity and individual personality that would require specific respect or consideration. It particularly suggests that they can and should be excluded from communication, interaction, and decision making since they have nothing meaningful to contribute and are ultimately not even persons with an own subjective perspective and volitional capacity anymore. By contrast, a life course perspective that follows the continuous development of the individual over time rather directs attention to remaining expressions and manifestations of personhood and volition in terms of biography, embodiment, and relationality.

In general, such a perspective promotes a view of dementia care in which the biography of the person concerned and their remaining strengths, abilities, and roles are given special weight. It must be ensured that people with dementia are empowered to participate in decisions about their daily lives. This means avoiding approaches that reinforce the perception of dementia patients as living corpses without a mind or a soul. The “zombification” of people with dementia disregards their moral status and their (still existing) autonomy and can effectively cause harm. It promotes stigmatising, discriminatory, and dehumanizing views and attitudes framing people with dementia as “post-persons” who have somehow no moral standing and no legal rights (Behuniak, 2011). This contradicts and undermines a person-centred and resource-oriented approach that emphasizes strengths rather than deficits, takes seriously the individual life history, personality, and needs and preferences of those affected, and is known to increase their wellbeing (Clarke et al., 2003).

Finally, our reflections also have theoretical implications for philosophical and ethical reflection in biomedicine and the life sciences. In particular, they sensitise us to the moral significance of biographical perspectives and the defining and framing influence of cultural patterns of interpretation. The main strands of contemporary bioethical reasoning are often based on an ageless and timeless image of the moral agent which is tacitly oriented towards the independent and rational individual of middle adulthood. Deviations from this image are therefore easily perceived as special developments or even abnormal and deficient phenomena (Jecker, (Fuchs 2020) ; Holm 2013). By contrast, a perspective that encompasses the entire human development from early childhood and adolescence to old age reveals that neediness and dependency are integral aspects of a normal life course that recur in different forms in different phases of life. Eventually, this may also reveal a deeper meaning of cultural metaphors that establish connections between dementia, childhood, or dying and death: They refer to fundamental existential dimensions of human experience that are difficult to articulate in conceptual terms and that could otherwise easily be ignored, marginalised, or at best expressed in an inappropriately abbreviated manner.

## Footnotes

1 Preliminary considerations on this topic were published in German in Schweda & Jongsma, 2018.
